# Supplementary material for: Understanding the Molecular Conformation and Viscoelasticity of Low Sol-Gel Transition Temperature Gelatin Methacryloyl Suspensions
Source: Int J Mol Sci. 2023 Apr 19;24(8):7489. doi: 10.3390/ijms24087489 (PMC10139010; doi:10.3390/ijms24087489)
Supplement: Supplementary file 1 [file ijms-24-07489-s001.zip › Supplementary Figure S1.pdf]

Supplementary materials

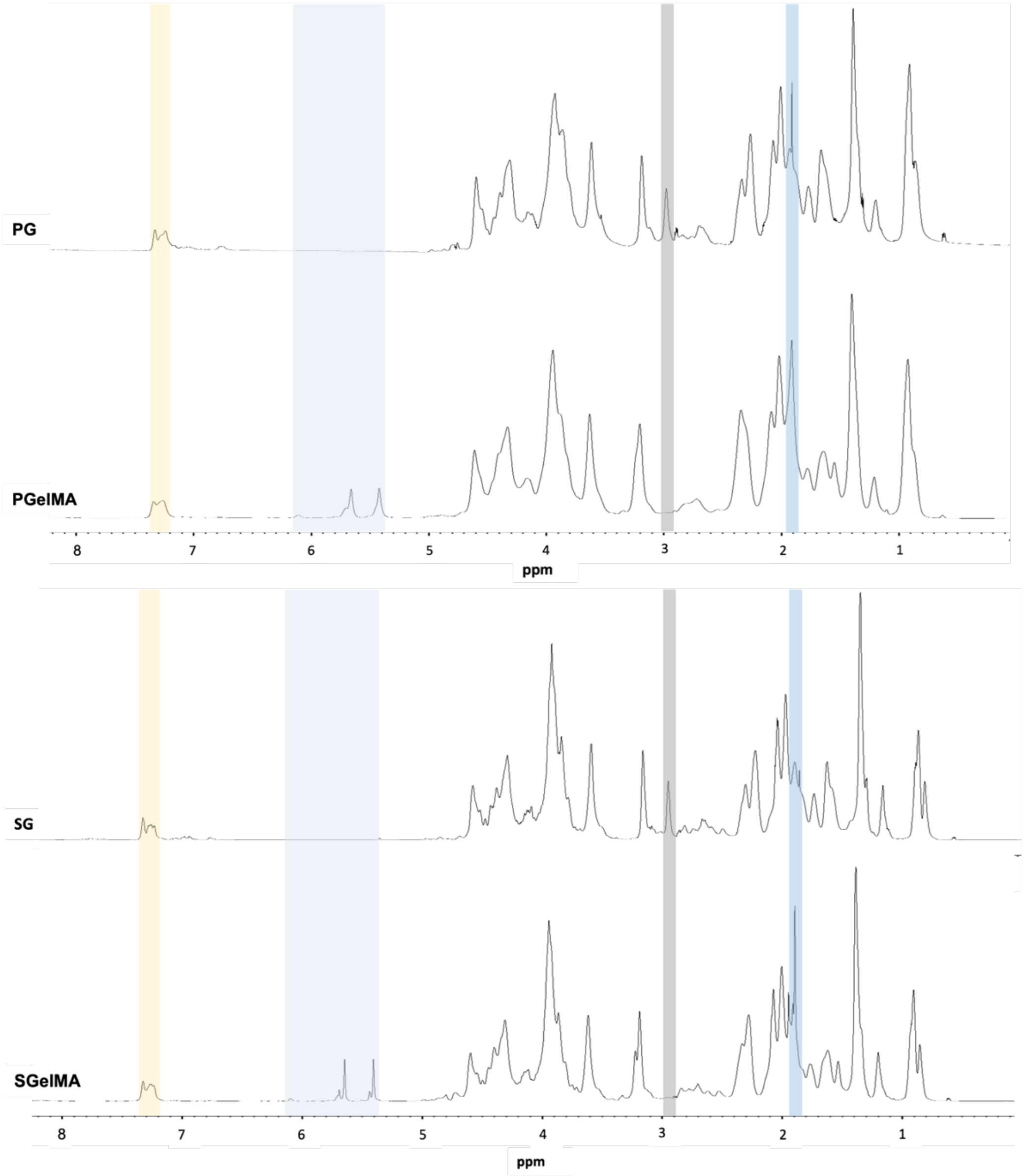

Figure S1. *1H-NMR spectra of gelatin and GelMA samples. Phenylalanine aromatic protons signal (7.20-7.40 ppm) (light yellow) was used for normalization, and lysine methylene signal (2.90-2.95 ppm) was used to determine GelMA degree of functionalization (in gray). New signals related to acrylic protons of methacryl groups (5.40-6.30 ppm) and methyl protons of methacryl groups (1.90 ppm) could be observed in spectra of GelMA from porcine and salmon origin (light blue and dark blue, respectively).*
