# Supplementary material for: Understanding the Molecular Conformation and Viscoelasticity of Low Sol-Gel Transition Temperature Gelatin Methacryloyl Suspensions
Source: Int J Mol Sci. 2023 Apr 19;24(8):7489. doi: 10.3390/ijms24087489 (PMC10139010; doi:10.3390/ijms24087489)
Supplement: Supplementary file 1 [file ijms-24-07489-s001.zip › Supplementary Figure S2.pdf]

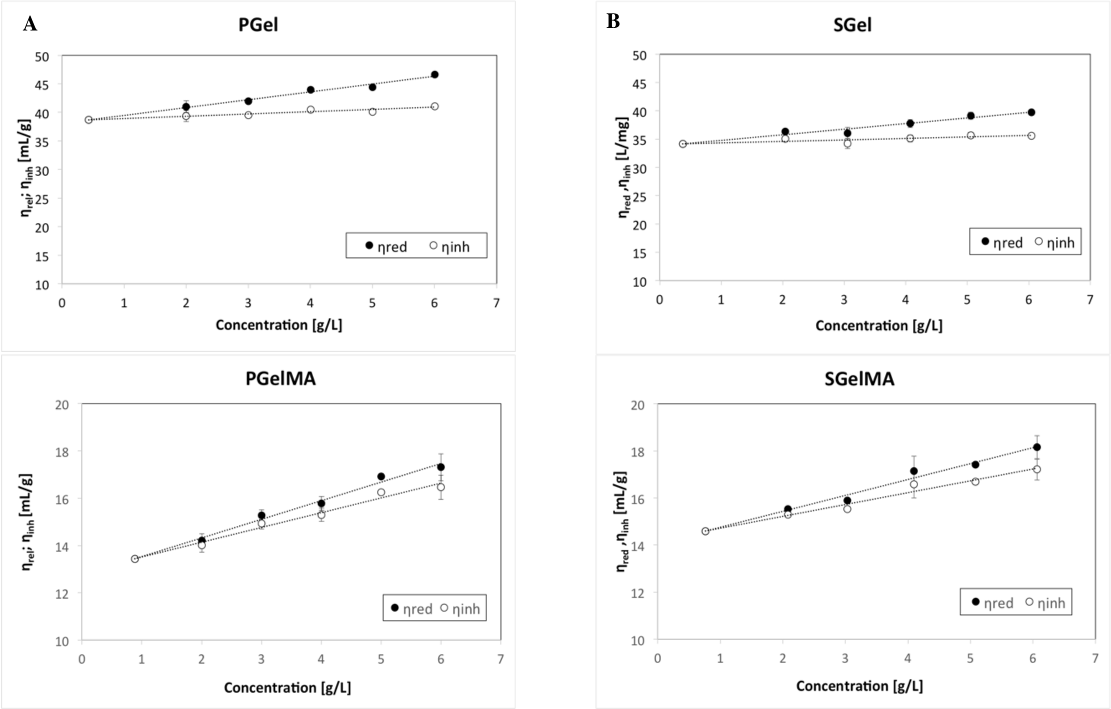

Figure S2. *Double extrapolation of the reduced viscosity (η_{red}) and inherent viscosity (η_{inh}) obtained by capillary viscometry, for the determination of the intrinsic viscosity [η]. A) PGel (top) and PGelMA (bottom), B) SGel (top) and SGelMA (bottom).*
