# Supplementary figures and images for: Understanding the Molecular Conformation and Viscoelasticity of Low Sol-Gel Transition Temperature Gelatin Methacryloyl Suspensions
Source: Int J Mol Sci. 2023 Apr 19;24(8):7489. doi: 10.3390/ijms24087489 (PMC10139010; doi:10.3390/ijms24087489)

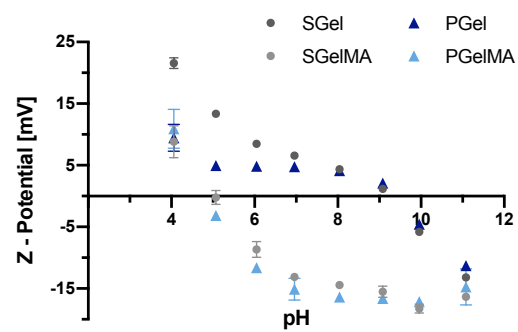

*Figure S3.  $\zeta$ -potential of different gelatin and GelMA suspensions at different pH values.*

Supplement: Supplementary file 1 [file ijms-24-07489-s001.zip › Supplementary Figure S3.pdf]

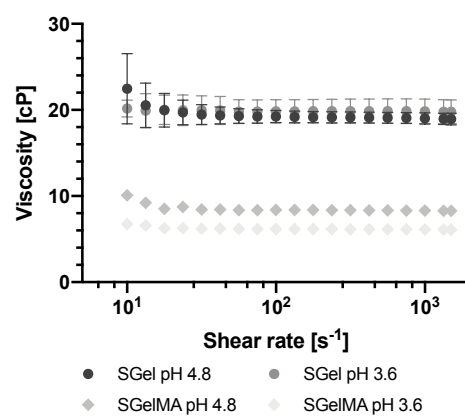

**Figure S4.** Shear rate and viscosity values of SGel and SGelMA suspensions (10% w/v) at pH ~ 3.6 and 4.8 at 20°C.

Supplement: Supplementary file 1 [file ijms-24-07489-s001.zip › Supplementary Figure S4.pdf]

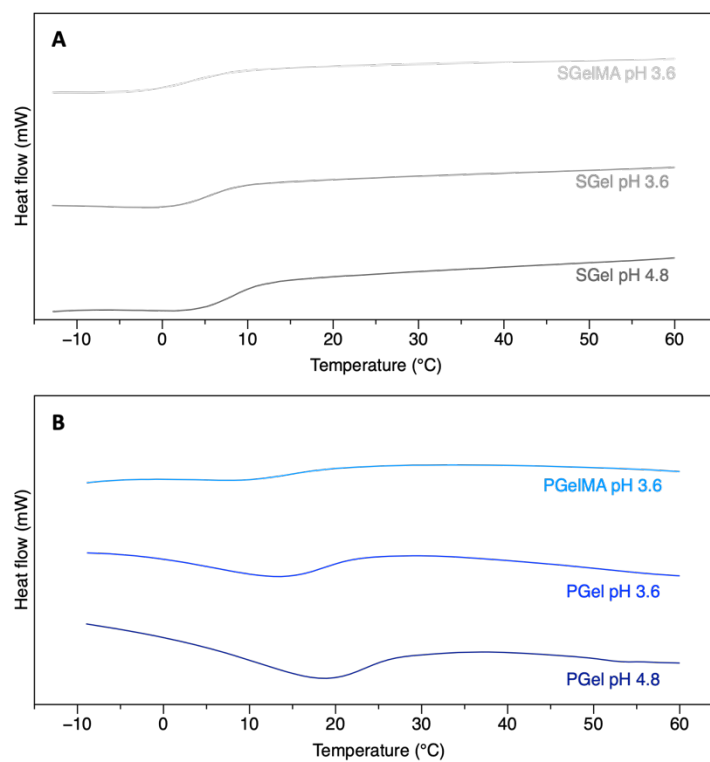

*Figure S5. DSC endotherms of gelatin and GelMA samples 20% w/v from salmon(A), and porcine (B) origin*

Supplement: Supplementary file 1 [file ijms-24-07489-s001.zip › Supplementary Figure S5.pdf]
